# Evaluation and associated factors of public health emergency management among medical college students in a city in Southwest China: a cross-sectional study

**DOI:** 10.1186/s12909-024-05317-1

**Published:** 2024-03-20

**Authors:** Xinrui Chen, Meng Zhang, Qingqing Bu, Bo Tan, Dan Deng

**Affiliations:** School of Public Health, Chongqing Medical University, Chongqing, China

**Keywords:** Medical college, Public health emergency, Management evaluation, Associated factors

## Abstract

**Background:**

Colleges and universities face an increased risk of public health emergencies. Among them, medical colleges and universities deserve more attention as they serve as the play a fundamental role in providing public health emergency services and in cultivating medical professionals. Effectively managing infectious disease prevention and control, as well as responding to public health emergencies in medical colleges and universities, is of great importance for enhancing the capacity of social emergency governance and improving the completeness of the public health system. This study aims to understand the management of public health emergencies in medical colleges in a city in southwest China, explore the factors associated with medical college students**’** evaluations, and provide recommendations for emergency management mechanisms in colleges and universities.

**Methods:**

In total, 781 medical college students were selected through stratified random sampling and surveyed using a questionnaire. The main factors affecting students**’** evaluation of emergency management were analyzed using multiple linear regression and structural equation modeling.

**Results:**

The overall emergency management situation in medical colleges was relatively complete, with satisfactory results. Medical college students**’** satisfaction with the timeliness of prevention measures was the highest, while the publicity and education were the lowest. Multiple linear regression analysis showed that grades, emergency education, -simulation training, -information reporting, and dynamic adjustment measures were associated with the evaluation of emergency management by medical students.

**Conclusions:**

Although the evaluation of emergency management in medical colleges was generally positive, certain limitations still existed. To improve the development of the public health system, colleges and universities should constantly reform and innovate emergency management mechanisms according to the important links in the prevention and control processes.

## Background

The construction of healthy China requires prioritizing people’s health, and effectively doing a good job in the prevention and control of infectious diseases and the response to public health emergencies [1]. A public health emergency refers to the sudden occurrence of severe infectious diseases, rapidly spreading diseases with unknown causes, widespread food and occupational poisoning, and other events or natural disasters threatening public health [2, 3]. Public health emergencies, especially outbreaks of infectious diseases, have become increasingly frequent. Recent public health emergencies have included severe acute respiratory syndrome (SARS) in 2003, influenza A (H1N1) in 2010, avian influenza A(H7N9) in 2013, and Corona Virus Disease 2019 (COVID-19); [4, 5] all posed a serious threat to global human health, economic development, and social stability [6, 7]. Southwest China has a large population, abundant biodiversity, a hot and humid climate, and a complex terrain These characteristics create favorable conditions for the emergence and spread of epidemiological threats to public health. Thus, it is imperative that this region of China strengthens its prevention and control measures for managing public health emergencies [8].

Colleges and universities are characterized by a large population density, frequent personnel turnover, the presence of shared public spaces, and high social attention, thereby placing them at an increased risk for public health emergencies [9]. Previous research has found that more than 70% of public health emergencies in China have occurred in schools; more than 80% were infectious disease epidemics or food poisoning incidents [10]. These emergencies considerably disrupted the schools’ operations and adversely affected the mental health of teachers, students, and other personnel, and also posed challenges with respect to the improvement of emergency plans and management mechanisms for responding to public health emergencies [11]. Other studies show that emergency prevention and control challenges remain for some colleges and universities, such as administrators’ insufficient attention, students’ inadequate understanding, poor medical facilities, professionals’ lack of coping skills, and imperfect information reporting systems [12–14], which render the emergency management of public health emergencies in colleges and universities difficult. Therefore, it is particularly important to strengthen emergency service guarantees for students, standardize internal emergency prevention and control management on campuses, and develop more effective strategies for dealing with public health emergencies.

At present, research on managing public emergencies in universities in other countries is gradually developing in the direction of systematization, specialization, and qualitative analysis [15]. However, the theoretical research on emergency management in China’s colleges and universities started late; most existing research focuses on descriptive research and case studies, and they have revealed the lack of comprehensive and evidenced-based management systems. Thus, their discussions of prevention and control management show a certain one-sidedness and sundry limitations [16]. In addition, although international research on the topic is relatively mature, the different national conditions of each country mean that their findings, while insightful, are not fully generalizable to China’s colleges and universities. Therefore, it is imperative to explore management strategies applicable to China when responding to public health emergencies.

Medical colleges and universities play a fundamental role in providing public health emergency services and in cultivating medical professionals [17]. The administrators of medical colleges and universities must ensure the safety and stability of students’ education by normalizing, standardizing, and specializing the schools’ emergency management. This requires timely identification of weak links in their emergency-response implementation and recommending improvements [18]. However, few studies have offered specific suggestions for public health emergency management in medical colleges and universities in China [19]. Students are the direct beneficiaries of public health emergency management systems in colleges and universities, and medical students have relevant professional theoretical knowledge and practical skills; therefore, they have professional advantages in dealing with public health emergencies [20]. Assessing the degree of satisfaction among medical students regarding emergency management in colleges and universities can facilitate the exploration of strategies to optimize public health emergency management in educational institutes. Therefore, this study aimed to investigate medical colleges’ management of public health emergencies in a city in Southwest China, systematically summarizing their emergency prevention and control implementation and their prevention and control achievements.

The study had several goals. First, it surveyed medical students’ satisfaction levels regarding schools’ emergency management and control measures. Second, it explored factors associated with their evaluations of the schools’ early prevention management, mid-crisis interventions, and late-stage security management. Finally, it re-examined some previously identified shortcomings in medical college and university emergency prevention and control for public health emergencies. The findings of this study may help Southwest China’s medical colleges and universities develop comprehensive, effective emergency management systems.

## Methods

### Study design and participants

The researchers used stratified random sampling to recruit students from several medical colleges and universities in one city in Southwest China. Each school was stratified by grade, and students with different majors were randomly selected from each grade. The study commenced only after the researchers communicated and coordinated with the schools’ administrators and obtained written informed consent from the participating students. The students completed a electronic questionnaire developed in line with the study’s objectives and issued through the schools’ student affairs offices, the survey period is March to April 2022. Of the 803 questionnaires collected, 781 were deemed valid, yielding an response rate of 97.26%. This study, which involved human subjects, was reviewed and approved by the Ethics Committee of Chongqing Medical University. And all methods were performed in accordance with the relevant guidelines and regulations.

### Quality control

To ensure the authenticity of the questionnaire, quality control measures were implemented. (1) Through preliminary literature research and expert consultation, based on the current situation of emergency management in colleges and universities and the policy direction of the National Health and Health Commission, the questionnaire preparation was completed and pre-investigation was conducted. Prior to the investigation, project investigators were trained. (2) The training for investigators emphasized the importance of introducing the purpose and significance of the study to participants before conducting the survey. Additionally, the training highlighted the need to clarify the requirements for filling out the questionnaire and any precautions that should be taken. And the survey should be completed anonymously to ensure the confidentiality of the participants’ responses. (3) Questionnaires requiring the same IP address could only be answered once. (4) After the questionnaire was submitted, questionnaires with any missing items and missing or unqualified basic information were deemed invalid.

### Questionnaire

The questionnaire contents were designed to satisfy the principles of relevance, universality, applicability and non-inductivity [21]. The survey included basic demographic information, the current situation as implemented, and medical students’ opinions on the schools’ prevention and control measures for public health emergencies. Seven items were used to evaluate the student’s views. The items covered publicity and education; emergency measures; daily monitoring; aftercare work; timeliness of announcements; timeliness of prevention and control measures; timeliness of administrators’ responses to public opinions The participants ranked each item using a five-point Likert scale from “1 = very dissatisfied” to “5 = very satisfied” for a total score of 35. The higher the score, the more satisfied the students. When the reliability and validity of the scale were assessed, Cronbach’s α coefficient was 0.930, the KMO validity coefficient was 0.920, and Bartlett’s test of sphericity *p* was < 0.001. Thus, the scale was considered to have good reliability and validity.

### Statistical analysis

Data were analyzed using IBM SPSS Statistics for Windows, Version 25.0, and Amos Version 24.0. The count data were described using percentages, and the satisfaction score were presented as (‾*x ± s* ). Mann-Whitney *U* test and Kruskal-Wallis *H* test were used to measure differences in quantitative data with non-normal distribution. Multiple linear regression and structural equation modeling were used to analyze the factors associated with medical students’ evaluations of public health emergency management (α = 0.05).

## Results

### Descriptive statistics

Among the 781 medical students, 227 (29.07%) were male, and 554 (70.93%) were female; 327 were freshmen (41.87%), 245 sophomores (31.37%), 174 juniors (22.28%), and 35 seniors or above (4.48%); 739 were undergraduate students (94.62%), and 42 were junior college students (5.38%).

### Medical colleges’ emergency responses to public health emergencies

When asked about publicity and education, 60.2% of the students said their school conducted emergency-related publicity and education once every six months. On emergency drills, 46.6% said their schools conducted emergency drills for public health incidents once a year. On emergency facilities, more than half of the students stated their school had public health facilities and conspicuously posted health safety signs. On preventive measures, more than 90% of the students reported their school had taken emergency preventive measures such as screening personnel for infections; controlling personnel activities; providing epidemic prevention materials; monitoring and reporting daily health conditions; and disinfecting key areas. On the administrators’ response to public opinions, 92.7% said their school issues timely responses and reports and informed the campus about the preventive measures, and 87.8% reported their schools regularly corrected disinformation and clarified unconfirmed statements. On emergency supplies, 97.8% of students stated their school distributed masks, disinfectants, thermometers, and other supplies. On aftercare counseling, 91.2% of the students stated their school provided mental health counseling and coping strategies.

### Medical colleges’ public health emergency prevention and control

The results showed that because of their school’s emergency management, the medical students reported their awareness of public health emergency prevention had improved greatly (60.1%), moderately (34.2%), or slightly (5.0%); only 0.8% thought it had not improved. Further, 58.6% thought that their school’s emergency prevention and control management ability had improved greatly compared to before the COVID-19 pandemic, 33.2% thought it had moderately improved, and 7.6% thought it had improved slightly; only 0.7% thought it had not improved.

### Medical students’ satisfaction with their schools’ management of public health emergencies

The students rated their satisfaction with their schools’ publicity and education, emergency measures, daily monitoring and management, aftercare work, timeliness of announcements, prevention and control measures, and the administrators’ responses to public opinions. The mean total score of their satisfaction ± standard deviation (*SD*) was 31.38 ± 4.06. The mean satisfaction score for the timeliness of the schools’ prevention and control measures was the highest (4.60 ± 0.60); the mean satisfaction score for publicity and education on food safety, laboratory safety, or epidemic prevention and control in schools was the lowest (4.31 ± 0.76). According to the single factor difference analyses (the Mann-Whitney *U* test and the Kruskal-Wallis *H* test), there were intergroup differences in the satisfaction scores of different grades and schools regarding the need for the following: emergency education, emergency drills, information notification, dynamically adjusted prevention and control measures, public opinion responses, and psychological counseling, which were all statistically significant (*p* < 0.05). Table 1 presents these results.

**Table 1:** Difference analysis of satisfaction scores of emergency management in medical colleges

Characteristics	Score	Z/H	*p*-value
**Gender**			
Male	31.61 ± 4.10	-1.858	0.063
Female	31.28 ± 4.04		
**Grade**			
Freshman	32.08 ± 3.79	20.889	< 0.001
Sophomore	30.98 ± 4.09		
Junior	30.80 ± 4.34		
Senior year and above	30.49 ± 4.00		
**Whether the school carried out emergency education**			
Yes	31.53 ± 3.93	-3.952	< 0.001
No	28.63 ± 5.21		
**Whether the school carried out emergency drills**			
Yes	31.67 ± 3.91	-5.783	< 0.001
No	29.31 ± 4.46		
**Whether the school had public health facilities**			
Yes	31.42 ± 4.02	-0.684	0.494
No	29.53 ± 5.19		
**Whether the school carried out information notification**			
Yes	31.55 ± 3.96	-4.661	< 0.001
No	30.12 ± 4.51		
**Whether the school dynamically adjusted the prevention and control measures**			
Yes	31.45 ± 4.00	-4.309	< 0.001
No	28.06 ± 5.14		
**Whether the school distributed emergency materials**			
Yes	31.53 ± 3.93	-1.554	0.120
No	28.63 ± 5.21		
**whether the schools rapidly responded to public opinions**			
Yes	31.67 ± 3.91	-2.985	0.003
No	29.31 ± 4.46		
**Whether the school provided psychological counseling**			
Yes	31.42 ± 4.02	-3.027	0.002
No	29.53 ± 5.19		

### Analysis of the factors influencing the medical students’ evaluations

#### Multiple linear regression

The independent variables in the regression were factors with statistical significance in the univariate analysis; the dependent variable was the satisfaction score for emergency management in medical colleges. The results showed that the main influencing factors were grade; whether the school carried out emergency education, emergency drills, and information notification; and whether the school dynamically adjusted prevention and control measures (*p* < 0.05). Compared to the students in the lower grades, the students in the higher grades were less satisfied with the schools’ overall emergency management; and the higher the emergency management evaluation of the university carrying out emergency education, emergency drills, information notification, and dynamically adjusting the prevention and control measures. Furthermore, according to the standardized regression coefficient, whether the school carried out information notification had the greatest impact on the evaluations, followed by whether the school carried out emergency drills. Table 2 presents these results.

**Table 2:** Multivariable analysis of satisfaction scores of emergency management in medical colleges

Variables	B	SE	t	*p*-value	Tolerance	VIF
Grade						
Freshman						
Sophomore	-1.101	0.324	-3.401	0.001	0.829	1.206
Junior	-1.130	0.360	-3.141	0.002	0.834	1.199
Senior year and above	-1.511	0.683	-2.211	0.027	0.935	1.070
Whether the school carried out emergency education	1.336	0.669	1.996	0.046	0.838	1.193
Whether the school carried out emergency drills	1.680	0.450	3.730	< 0.001	0.847	1.180
Whether the school carried out information notification	3.964	0.777	5.100	< 0.001	0.895	1.117
Whether the school dynamically adjusted the prevention and control measures	1.834	0.627	2.924	0.004	0.893	1.119
Constant term	23.750	0.959	24.765	< 0.001		

SE = standard error; F = 15.114, *p* < 0.001, R^2^ = 0.120, D-W = 2.013. The tolerance of all independent variables was close to 1, and the variance inflation factor (VIF) was less than 10, so there was no multicollinearity

#### *Structural equation model*

**Fig. 1:**
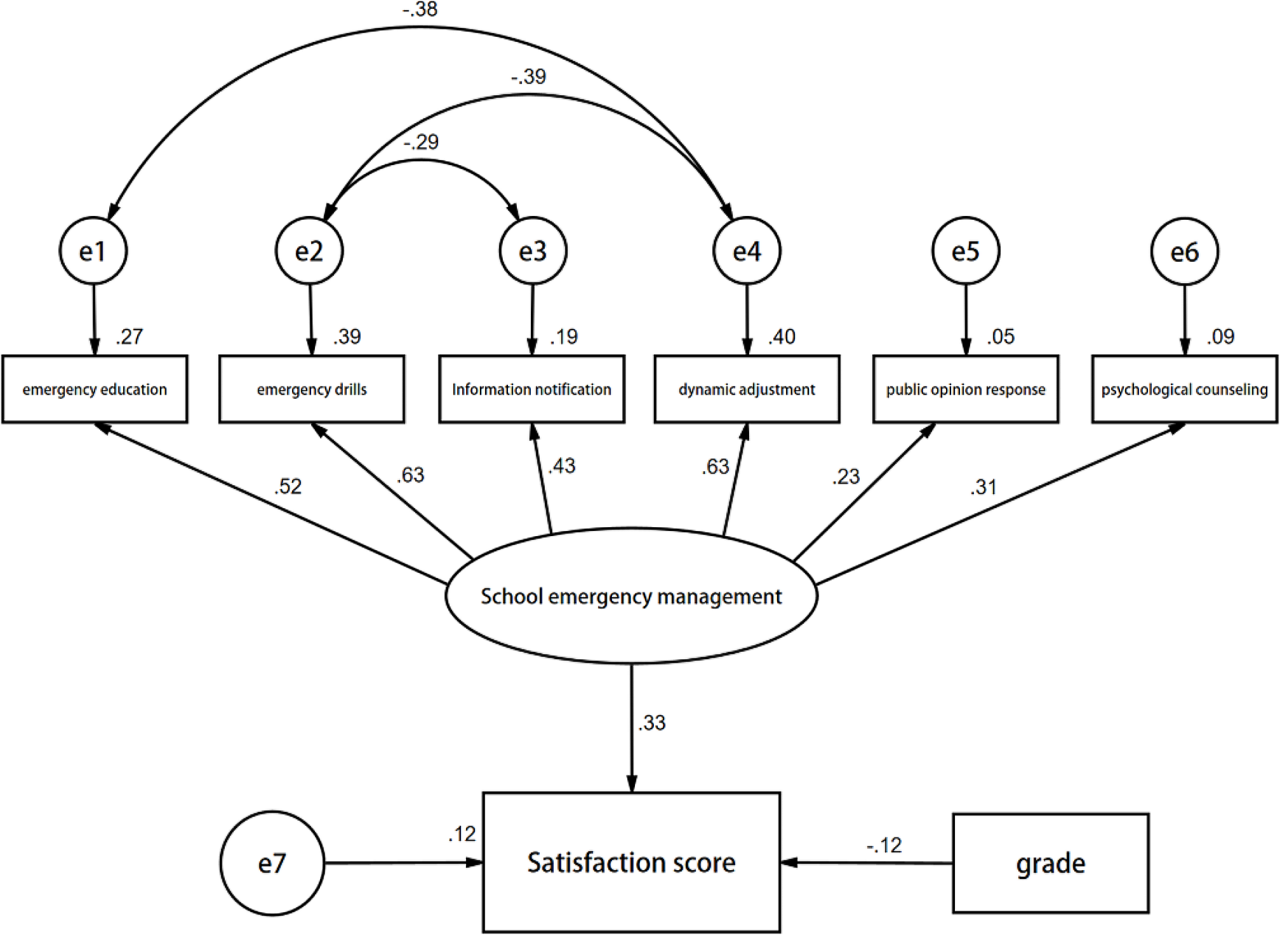
Structural equation model analysis of emergency management evaluation in medical colleges

The researchers used structural equation modeling (SEM) to explore the determinants of the medical students’ evaluations of their schools’ emergency management and the relationship between these factors. The SEM model included these variables: grade; whether the school carried out emergency education, emergency drills, or information notification; whether the school dynamically adjusted its prevention and control measures; whether the schools rapidly responded to public opinions; whether the school provided psychological counseling; and the students’ satisfaction scores. The SEM model used these factors to explore the influences on the students’ evaluations of the public health emergency management of their colleges and universities and the correlation among the associated factors. Figure 1 shows an SEM path analysis diagram.

The model showed that grades and the schools’ emergency management measures directly impacted the students’ evaluations (*p* < 0.001). The standardized path coefficients were − 0.12 and 0.33, respectively. When the other variables were constant, the grades negatively influenced the evaluations, and the schools’ emergency management measures positively influenced the evaluations. These results aligned with the results of the multiple linear regression. In addition, the fitness indices met the requirements of the test statistics. Table 3 summarizes the results.

**Table 3:** Structural equation model fitness table

	Test statistic	Adaptation value	Degree of fit	Model-fitting judgment
Absolute fitness index	χ^2^/df	< 3.000	2.156	Coincidence
	GFI	> 0.900	0.988	Coincidence
	AGFI	> 0.900	0.975	Coincidence
	RMSEA	< 0.080	0.038	Coincidence
Value-added fitness index	NFI	> 0.900	0.859	Acceptable
	TLI (NNFI)	> 0.900	0.860	Acceptable
	IFI	> 0.900	0.919	Coincidence
	CFI	> 0.900	0.915	Coincidence

GFI = goodness-of-fit index; AGFI = adjusted goodness-of-fit index; RMSEA = root mean square error of approximation; NFI = normed fit index; TLI = Tucker–Lewis index (also called non-normed fit index or NNFI); IFI = incremental fit index; CFI = comparative fit index

## Discussion

Understanding what influences medical students’ evaluations of their schools’ public health emergency management and responses could help colleges and universities in China improve their emergency management strategies [22] This study found that the satisfaction score of the timeliness of prevention and control measures taken by schools was the highest, indicating that the city’s medical colleges quickly implemented appropriate epidemic prevention policies and instituted timely and effective prevention and control measures to avoid the spread of COVID-19; thus, they would respond equally well with other epidemics and public health emergencies. Xu and Chen [23] also found that the timeliness of schools’ emergency measures effectively improved college students’ sense of security. Although more than half of the students said that their school carried out emergency education on food safety, laboratory safety, or epidemic prevention and control once every six months, the lowest satisfaction scores were for the schools’ emergency education, indicating that the medical colleges and universities lacked targeted, systematic, and universal education systems while ensuring the quantity of education, resulting in poor quality and insufficient epidemiological information. These findings align with Liu [24].

The students’ grades and the schools’ implementation of emergency management measures were the main factors associated with students’ evaluations. The students in the higher grades were less satisfied with the schools’ emergency management, and the difference was statistically significant. Zhu and Zhang [25] also confirmed this conclusion. They believe that the proportion of senior students who were confident of coping with disasters is not very high, and the proportion who believe that disaster education is beneficial to coping with disasters is also not very high. The reason might be that emergency-related education is mainly handled during freshmen enrollment, and with the deepening of professional understanding, the demand and expectation of senior students to take emergency management measures is increasing gradually. The schools had higher evaluation scores for emergency drills, epidemic prevention information, and the dynamic adjustment of prevention and control measures, consistent with Wang [26]. As emergency education can increase students’ emergency knowledge reserve, emergency drills can boost their emergency prevention and rapid response skills. Timely, comprehensive, and dynamically adjusted prevention and control measures and information can mitigate emergencies’ inconveniences and adverse effects, increasing students’ trust in the schools’ emergency management competence.

The study’s results showed that emergency prevention and intervention measures are critical in managing public health emergencies in colleges and universities, as reported by Li [27]. The administrations of medical and other colleges and universities should actively implement and improve campus emergency response systems. These systems should consider the factors influencing students’ evaluations, to ensure the continuous improvement of the schools’ prevention and control management strategies of public health emergencies, as follows.

1) *Provide comprehensive education on preventing and handling public health emergencies* [28]. Medical school administrations must establish a reasonable emergency education system; regularly provide high-quality, understandable, science-based information and training on epidemic prevention and control to the medical students at every level of study, especially the seniors; and ensure campus-wide compliance with posted guidelines on public health safety regulations.

2) *Have professionals conduct regular, standardized public health emergency drills* [29]. Colleges and universities should have professionals on-site trained in managing public health emergencies, Regularly carrying out emergency drills under the guidance of the local party committee and the government, and instructing students, teachers, and staff about emergency-response topics such as remote classes; campus closures or restrictions; health-related isolation and treatment; health data collection, sorting, and analysis; epidemic investigation and tracing; and regional detection and quarantines to expedite emergency response.

3) *Dynamically respond to the needs of students, teachers, and the staff during a public health crisis.* Public health emergency underscored the need for rapid, flexible, accurate communication and control strategies. Universities and colleges should provide crisis-related information; prevention, transmission, and treatment guidance; and up-to-date news and official school announcements. It is vital to keep everyone informed about everything from the nature of the emergency to teaching adjustments, such as temporarily transitioning to remote learning [30].

4) *Ensure the students’ physical and mental well-being.* Colleges and universities should anticipate how public health emergencies can adversely affect students’ physical and mental health [31]. They should discuss this in medical students’ classes—before, during, and after a health crisis—and provide mental health education and psychological counseling.

5) *Respond honestly and swiftly to questions and concerns.* Misinformation and the lack of information can exacerbate public health emergencies [32]. Medical school administrators should establish mechanisms for handling the public’s questions and concerns by building effective communication channels between colleges and students.

### Limitations

The index system used in this study should be improved further, more research indicators can be included according to the specific conditions of different university types. And this study was a cross sectional design, it was aimed to explore the association between certain variables and the outcomes of interest, rather than to establish a causal relationship. In addition, the use of self-reported data may introduce some limitations to the study, as students may have answered the questions as they felt required by the university or government, rather than reflecting their true perceptions. To mitigate potential response bias and ensure validity and reliability, we conducted repetitive questionnaire designs and pilot experiments.

## Conclusion

This study evaluated the factors influencing medical students’ satisfaction with their schools’ emergency management systems and responses in a city in Southwest China and examined the existing problems in emergency prevention and control of public health emergencies in colleges and universities. Students’ grades and the implementation of emergency management measures in schools were important factors affecting the evaluation of emergency management of public health emergencies in colleges and universities. Since today’s medical students will be tomorrow’s professionals entrusted with responding to public health emergencies, improving the medical schools’ management strategies for public health emergencies is vital.

## Data Availability

The datasets used and/or analysed during the current study are available from the corresponding author on reasonable request.

## Abbreviations

COVID-19: Corona Virus Disease 2019
IP address: Internet protocol address
SARS: Severe acute respiratory syndrome
SEM: Structural equation modeling
